# Evidence for massive methane hydrate destabilization during the penultimate interglacial warming

**DOI:** 10.1073/pnas.2201871119

**Published:** 2022-08-22

**Authors:** Syee Weldeab, Ralph R. Schneider, Jimin Yu, Andrew Kylander-Clark

**Affiliations:** ^a^Department of Earth Science, University of California, Santa Barbara, CA 93106;; ^b^Institute of Geosciences, Kiel University, 24118 Kiel, Germany;; ^c^Pilot National Laboratory for Marine Science and Technology (Qingdao), Qingdao 266237, China;; ^d^Research School of Earth Sciences, The Australian National University, Canberra, ACT 2601, Australia

**Keywords:** methane hydrate destabilization, oceanic intermediate water warming, Gulf of Guinea, meltwater-induced AMOC weakening, The Eemian

## Abstract

The stability of widespread methane hydrates in shallow subsurface sediments of the marine continental margins is sensitive to temperature increases experienced by upper intermediate waters. Destabilization of methane hydrates and ensuing release of methane would produce climatic feedbacks amplifying and accelerating global warming. Hence, improved assessment of ongoing intermediate water warming is crucially important, especially that resulting from a weakening of Atlantic meridional overturning circulation (AMOC). Our study provides an independent paleoclimatic perspective by reconstructing the thermal structure and imprint of methane oxidation throughout a water column of 1,300 m. We studied a sediment sequence from the eastern equatorial Atlantic (Gulf of Guinea), a region containing abundant shallow subsurface methane hydrates. We focused on the early part of the penultimate interglacial and present a hitherto undocumented and remarkably large intermediate water warming of 6.8 °C in response to a brief episode of meltwater-induced, modest AMOC weakening centered at 126,000 to 125,000 y ago. The warming of intermediate waters to 14 °C significantly exceeds the stability field of methane hydrates. In conjunction with this warming, our study reveals an anomalously low δ^13^C spike throughout the entire water column, recorded as primary signatures in single and pooled shells of multitaxa foraminifers. This extremely negative δ^13^C excursion was almost certainly the result of massive destabilization of methane hydrates. This study documents and connects a sequence of climatic events and climatic feedback processes associated with and triggered by the penultimate climate warming that can serve as a paleoanalog for modern ongoing warming.

## Significance

Our results identify an exceptionally large warming of the equatorial Atlantic intermediate waters and strong evidence of methane release and oxidation almost certainly due to massive methane hydrate destabilization during the early part of the penultimate warm episode (126,000 to 125,000 y ago). This major warming was caused by reduced advection of cold water from high latitudes and enhanced downward heat diffusion in response to a brief episode of meltwater-induced weakening of the Atlantic meridional overturning circulation and amplified by a warm mean climate. Our results highlight climatic feedback processes associated with the penultimate climate warming that can serve as a paleoanalog for modern ongoing warming.

Because ocean intermediate waters impinge on marine sediments that often contain potentially unstable shallow subsurface methane hydrates (1–3), better understanding is crucial about the factors that contribute to intermediate water warming and their potential extent, especially in context with ongoing global warming. Simulation studies have suggested warming of intermediate waters has been limited to ∼1.5 °C to 3 °C, and that such warmings were insufficient to significantly affect the stability of shallow subsurface methane hydrates (2–5). However, the magnitude of intermediate water warming can be significantly amplified by meltwater-induced weakening of atmospheric and ocean circulation (6–11), an amplification not considered in the simulations that examined potential gas hydrate destabilization (2–5). A recent simulation study estimates the contribution of weak Atlantic meridional overturning circulation (AMOC) at 0.3 °C to 0.4 °C to the warming of the intermediate waters for a business-as-usual scenario at the end of the 21st century (12), a modest contribution compared to observations in past climate studies (6–11). An accelerated mass loss of the Greenland ice sheet and the associated freshening of subpolar North Atlantic sea surface waters represents a robust proxy of ongoing rapid global warming (13). Causally linked to this freshening is increasing evidence of a steady weakening of the AMOC (14, 15). Warming of the intermediate waters by 3 °C to 5 °C in response to a meltwater-induced AMOC weakening is a robust feature of the last deglacial (6–11). This corresponds to pockmark formations on the ocean floor and extremely negative foraminiferal δ^13^C values in sediment sequences that reflect methane hydrate dissociation in response to intermediate water warming related to meltwater-induced weakening of AMOC and associated changes in atmospheric circulation during the last deglacial (10, 11, 16–18). A sequence of episodic, extremely low foraminiferal δ^13^C values observed in Late Quaternary sediments of Santa Barbara Basin led to the formulation of the “clathrate gun hypothesis” (10, 11). The hypothesis states that episodic warming of intermediate waters during the last glacial and early deglacial led to dissociation of shallow subsurface methane hydrates and release of methane, contributing to the observed increases of atmosphere methane concentrations, further contributing to climatic warming episodes (10, 11). The key findings of our study add to a growing body of observational findings strongly supporting the “clathrate gun hypothesis” (10, 11). The magnitude of intermediate water warming in response to AMOC weakening most likely is critically dependent on the existing mean climatic state. Importantly, the interval we have studied is marked by a mean climate state comparable to future projections of transient global climate warming of 1.3 °C to 3.0 °C (19). Our findings thus provide insights about major meltwater-induced intermediate water warming during warm episodes like the present with the potential to destabilize structural-type methane hydrates.

In this study, we focus on the early part of the Eemian interglacial episode (128,000 to 125,000 y before present [ky BP]), the youngest episode when tropical oceans were warmer than the Holocene by up to 2 °C (20–22). We show that the combination of a warm mean climate state and a relatively brief and modest episode of meltwater-induced AMOC weakening produced an exceptionally large intermediate water warming that significantly exceeds the stability field of methane hydrates. Coincident with this warming, we demonstrate that the dissolved inorganic carbon across the entire 1,300-m water column was marked by an anomalously low carbon isotope ratio (^13^C/^12^C) which we interpret to indicate destabilization of shallow subsurface methane hydrates and ensuing methane oxidation.

## Anomalously Low δ^13^C across the Entire Water Column

**Fig. 1.**
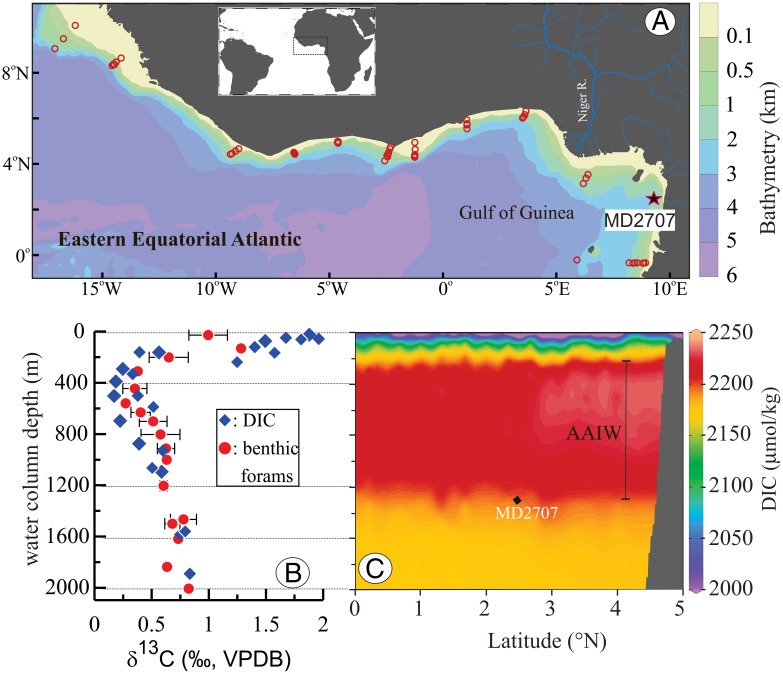
Setting of study area and the depth distribution of key environmental parameters. (*A*) Eastern equatorial Atlantic and Gulf of Guinea with the location of MD2707 sediment core (2°30.11'N, 9°23.68'E, 1,295-m seafloor water depth) and core-top samples indicated by a star and red open circles, respectively. (*B*) Blue diamonds: δ^13^C of DIC from the eastern tropical Atlantic (24°S to 11°N, 20°W to 6.46°E) (23). Red dots: zonally averaged and depth-binned (100 m) δ^13^C values of *Cibicides* spp. of core-top samples shown in *A* and *SI Appendix*, Table S1. Horizontal bars indicate SD from the mean of samples that are binned at an interval of 100-m water depth. (*C*) DIC plotted against water column depth in the Gulf of Guinea (7°W to 6°E/2°S to 6°N) (69). The depth water of Antarctic Intermediate Water (AAIW) extends between 300 and ∼1,200 m. The water depth and latitude of MD2707 is indicated by a black diamond.

The zonally averaged and depth-binned δ^13^C values of Rose Bengal–stained epibenthic foraminifers (*Cibicides* spp.) from numerous Gulf of Guinea core-top samples exhibit a systematic shift in δ^13^C values of dissolved inorganic carbon related to changes in water depth (23) (Fig. 1 and *SI Appendix*, Table S1). Importantly, waters between 200 and 1,200 m represent Antarctic Intermediate Water marked by relatively low δ^13^C values (0.25 to 0.58‰). In deeper water depths between 1,200 and 1,400 m, δ^13^C values are slightly higher (up to 0.8‰) and represent intermediate values between Antarctic Intermediate Water and North Atlantic Deep Water. These systematic depth changes in the δ^13^C composition of *Cibicides* spp. are valuable in providing a proxy for determining past changes in the sources of dissolved inorganic carbon which, in turn, reflect changes in ocean circulation and climate.

**Fig. 2.**
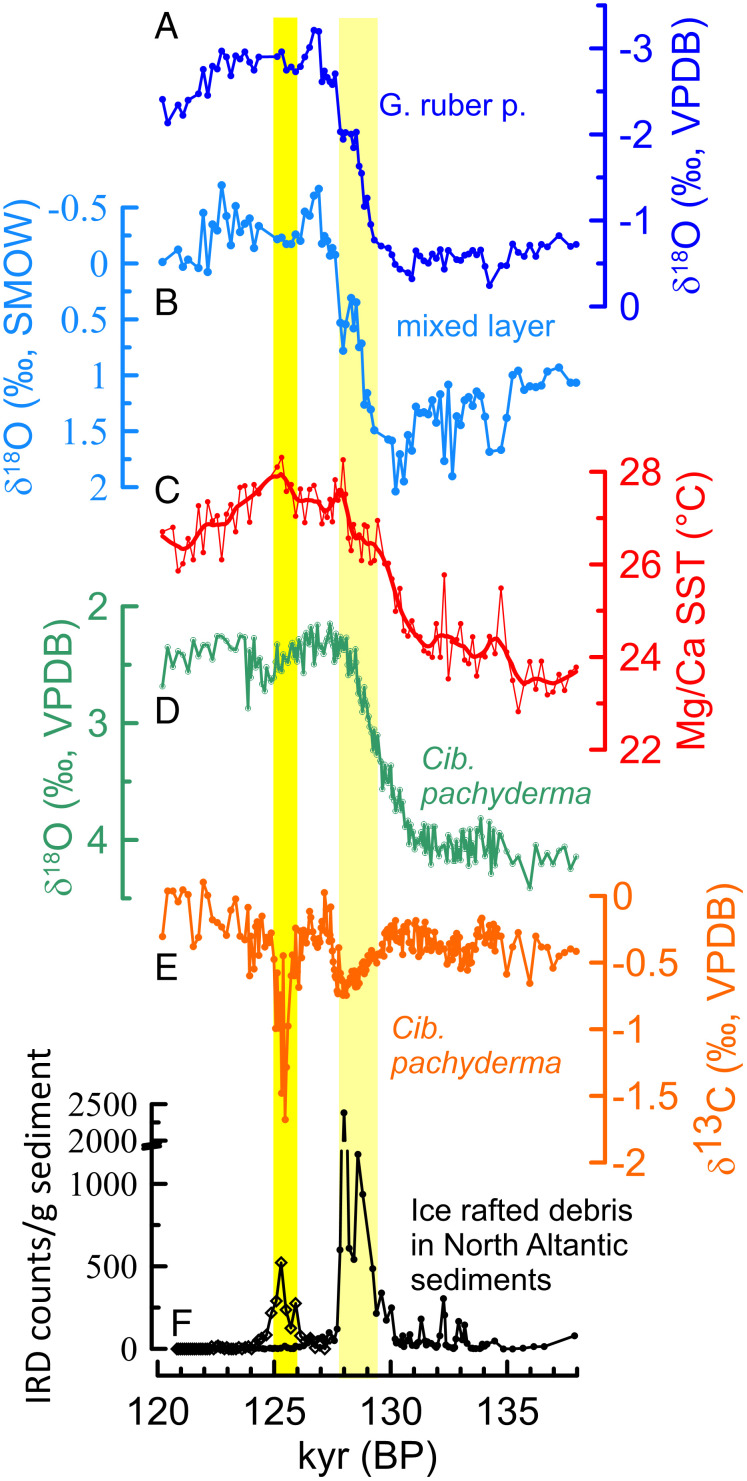
Proxy data from MD2707 core samples representing the time interval between 120 and 138 ky BP. (*A*) The δ^18^O time series analyzed in *G. ruber* pink, a mixed layer dwelling planktonic foraminifer. (*B*) The δ^18^O of mixed layer sea water and (*C*) sea surface temperature (SST) based on Mg/Ca and δ^18^O analyses in *G. ruber* pink (20). (*D* and *E*) The δ^18^O and δ^13^C time series analyzed in *C. pachyderma*, an epibenthic and shallow infaunal benthic foraminifer. (*F*) Records of IRD in North Atlantic sediments [open diamond (29) and dots (25)]. Yellow bands indicate increased IRD and possibly weakened AMOC centered around 129 and 125 ka.

**Fig. 3.**
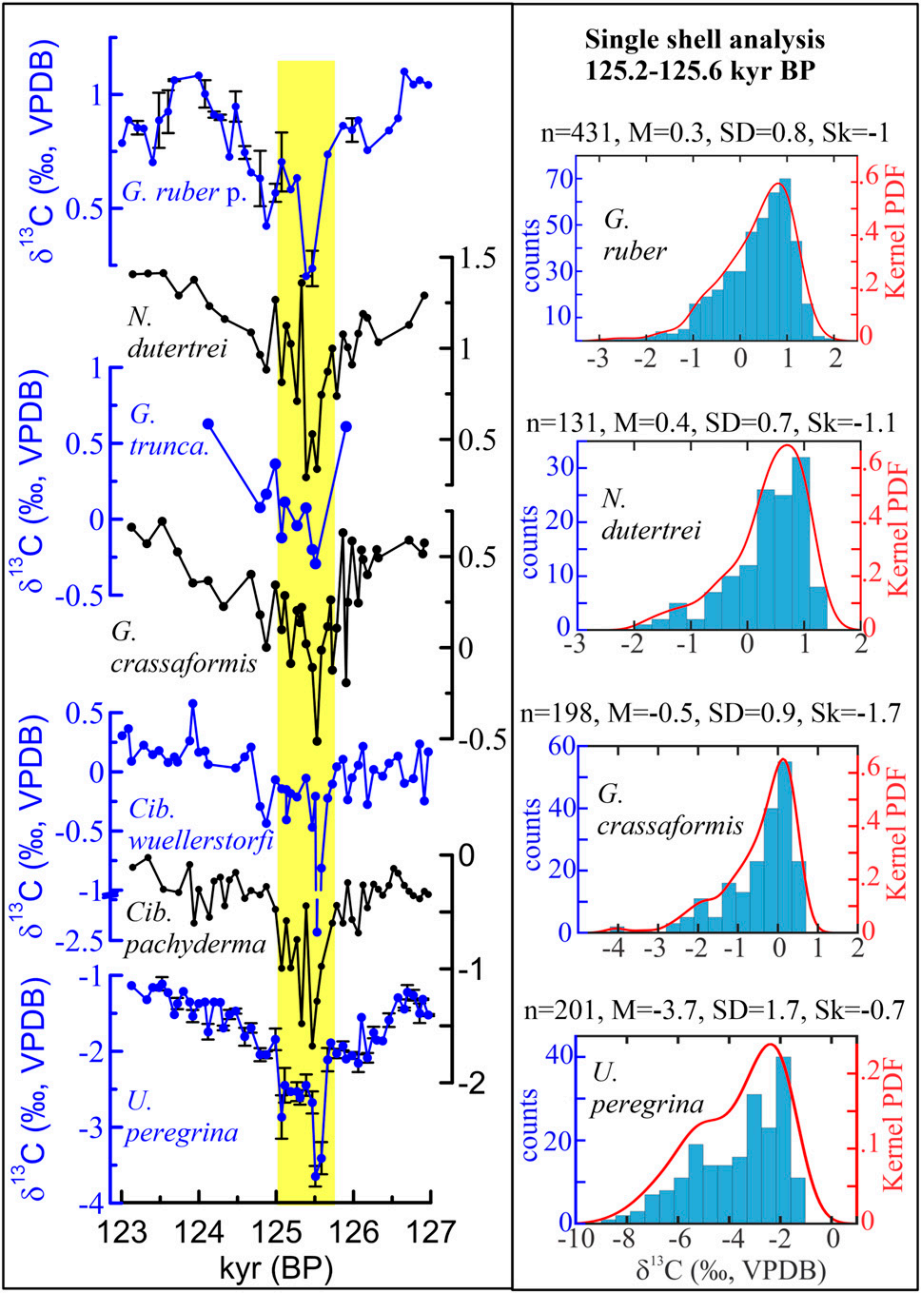
The δ^13^C analyses for pooled and single tests (shells) of benthic and planktonic foraminifers representing the interval between 123 and 127 ky BP. (*Left*) The δ^13^C for tests of *G. ruber* pink, a mixed layer (0 m to 25 m) dwelling plankton foraminifer; *N. dutertrei*, a mixed layer and thermocline (50 m to 115 m) dweller planktonic foraminifer; *G. truncatulinoides*, a thermocline/deep (∼400 m) dweller planktonic foraminifer; *G. crassaformis*, a deep (∼700 m) dweller planktonic foraminifer; *C. wuellerstorfi* (epibenthic); *C. pachyderma* (epibenthic and shallow infaunal); and *U. peregrina* (deep infaunal). Vertical bars in the *G. ruber* and *U. peregrina* data indicate SD from the mean of two or three repeat analyses. The yellow shaded area indiacates the time window of the negative carbon isotope anomaly. (*Right*) Histograms and kernel probability density functions (PDF) showing nonparametric and highly skewed distributions of δ^13^C analyzed in a single test of planktonic and benthic foraminifers in four or five samples from the time interval of 125.2 ky BP to 125.6 ky BP showing extremely anomalous δ^13^C values in the pooled isotope analyses on *Left*. The number of analyses (n), the mean value (M), SD from the mean (SD), and skewness (Sk) for each dataset are shown above the histograms.

**Fig. 4.**
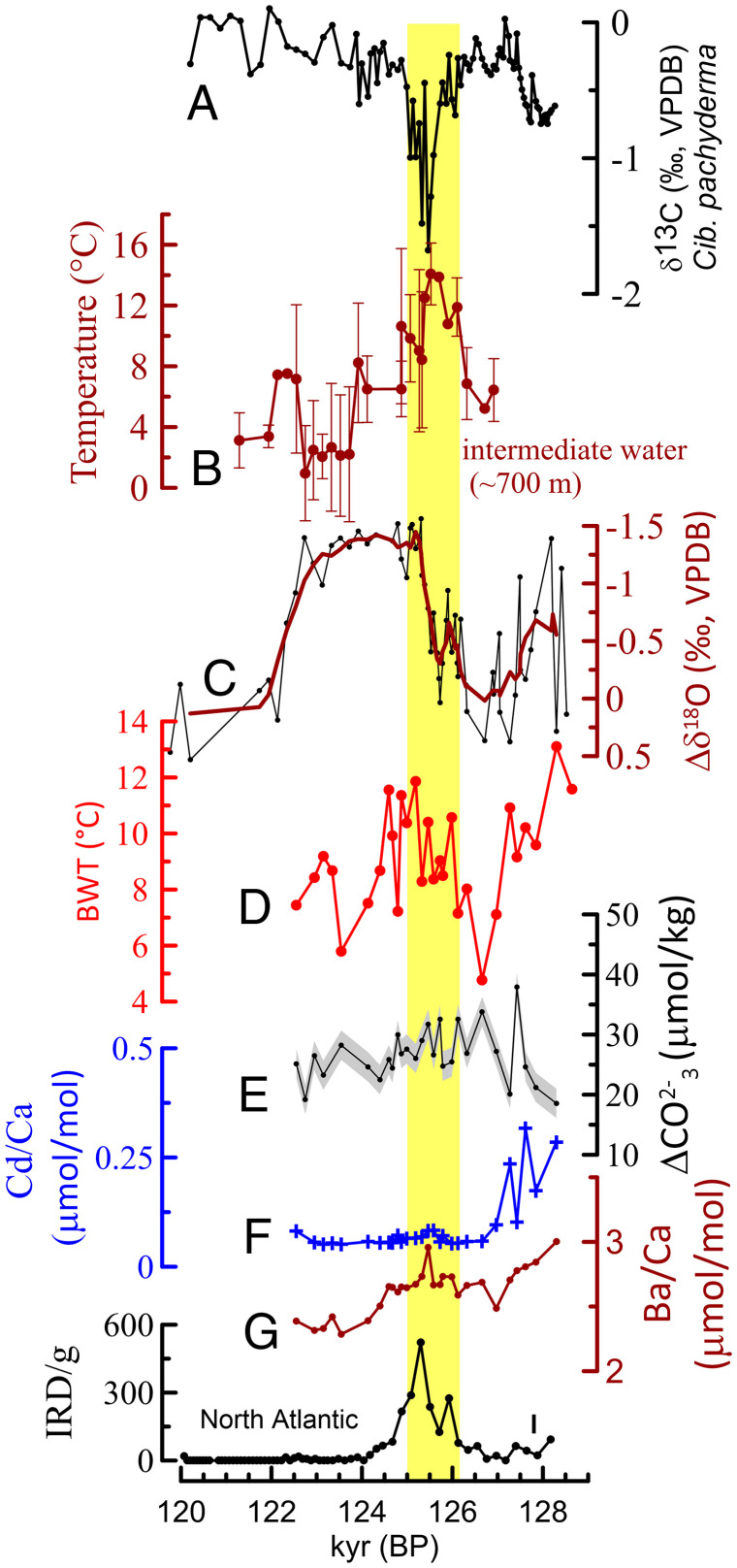
Changes in multiple proxies that show linkages between gas hydrate destabilization, warming of intermediate waters, and meltwater-induced weakening of the AMOC. (*A*) The δ^13^C of pooled *C. pachyderma* in MD03-2707. (*B*) Temperature reconstruction of intermediate water using Mg/Ca in *G. crassaformis* with a depth habitat of ∼700 m. (*C*) Changes in δ^18^O of *G. crassaformis* relative to the immediate interval before the anomalous decrease of δ^13^C. (*D*) BWT reconstruction based of Mg/Ca analysis in *C. wuellerstorfi*. (*E*) B/Ca-based (*C. wuellerstorfi*) estimates of carbonate ion concentration changes. (*F* and *G*) Cd/Ca and Ba/Ca values in tests of *C. wuellerstorfi*. (*I*) IRD recorded in North Atlantic sediment (29). The yellow shaded area indicates the time window of the carbon isotope anomaly.

During the early part of the penultimate warm episode and the preceding penultimate deglacial, the epibenthic/shallow infaunal benthic foraminifer (*Cibicides pachyderma*) δ^13^C time series (pooled records) reveals two conspicuous brief episodes marked by especially low δ^13^C values (Fig. 2). The first of these peaks occurred between 127.9 ± 1.3 and 129 ± 1.1 ky BP, with an average δ^13^C value of −0.66 ± 0.05‰ (*n* = 24). The second peak occurred between 126 ± 1.4 and 125 ± 1.3 ky BP, with values up to −1.68‰. Because of its especially prominent signature, we have focused on the second event (126 to 125 ky BP) by expanding our isotope analyses to include pooled tests of a number of taxa that lived in different depth habitats. These are *Uvigerina peregrina*, a deep infaunal benthic foraminifer; *Cibicides wuellerstorfi*, an epibenthic foraminifer; *Globorotalia crassaformis*, a deep-dwelling planktonic foraminifer with an average depth habitat of 700 ± 150 m; *Globorotalia truncatulinoides,* a planktonic foraminifer with a depth habitat of 400 ± 115 m; mixed-layer dwelling planktonic foraminifers *Neogloboquadrina dutertrei* (habitat depth: 115 m to 50 m) and *Globigerinoides ruber* pink (habitat depth: 0 m to 25 m) (24). This multitaxa record clearly exhibits a distinct trough with anomalously low δ^13^C values, and a strong and coherent water column gradient (Fig. 3) with the lowest δ^13^C value of −3.74‰ in *U. peregrina*, followed by −2.45‰ and −1.68‰ in *C. wuellerstorfi* and *C. pachyderma*, −0.51‰ in *G. crassaformis*, −0.29‰ in *G. truncatulinoides*, 0.29‰ in *N. dutertrei*, and 0.2‰ in *G. ruber*. In contrast, the δ^18^O time series exhibit variable trends between these same taxa (Fig. 4 and *SI Appendix*, Fig. S2). With an average δ^18^O value of 3.16 ± 0.1‰ (*n* = 17), *U. peregrina* exhibits comparable values (3.17 ± 0.1‰, *n* = 14) to those before the carbon isotopic anomaly. In contrast, δ^18^O increased by 0.25‰ in *C. pachyderma* compared with values before the anomaly, with a maximal value of 2.5‰. Contemporaneous with the onset of the carbon isotopic anomaly, the δ^18^O values significantly decreased in *C. wuellerstorfi*, *G. crassaformis*, and *G. truncatulinoides,* with the greatest decrease (1.9‰) exhibited in *G. crassaformis* relative to the values before the carbon isotope anomaly (Fig. 4 and *SI Appendix*, Fig. S2). *N. dutertrei* and *G. ruber* pink exhibit a decline of δ^18^O by up to 0.5‰ and 0.25‰, respectively (*SI Appendix*, Fig. S2).

Due to sediment bioturbation, reconstruction of such brief climatic events with a duration shorter than the bioturbation depth (∼10 cm) is recorded in the analyzed pooled samples as a composite of the background signal and the signal of the brief event itself. Because of this, to reconstruct the full extent of this brief event, we analyzed the δ^13^C and δ^18^O of single tests of benthic and planktonic foraminifers from selected samples within the episode of low δ^13^C values centered between 126 and 125 ky BP. The resulting δ^13^C data show a nonparametric distribution with a strong negative skewness (Fig. 3), while the corresponding δ^18^O is normally distributed, with the exception of *G. crassaformis* for which the δ^18^O values exhibit a negative skewness and a positive and significant correlation with the δ^13^C (*SI Appendix*, Figs. S2 and S3). The most negative δ^13^C values exhibited by these single specimens range up to −8.7‰ in *U. peregrine* (sediment pore water), −4.03‰ in *G. crassaformis* (intermediate water depth), −1.83‰ in *N. dutertrei* (thermocline/lower mixed layer dweller), and −2.8‰ in *G. ruber* (mixed layer dweller). These extreme values demonstrate brief, major changes in the source of the dissolved inorganic carbon (DIC) affecting the entire water column.

## Ruling Out Diagenetic Effects as a Cause for the δ^13^C Anomaly

The first low δ^13^C peak (−0.66 ± 0.05‰, *n* = 24) centered at ∼127.9 ky BP to 129 ky BP coincided with a distinct episode of atmospheric and ocean circulation change that included a weakening of West African and Asian monsoons, North Atlantic surface water cooling and freshening, and a weakened AMOC (Fig. 2) (20, 25–27). We consider the average δ^13^C value of this event as representing the effects of meltwater-induced ocean circulation changes. The second event centered between 126 ± 1.4 and 125 ± 1.3 ky BP not only is much more pronounced, but the anomalously low δ^13^C values were also clearly recorded throughout the entire water column (Fig. 3)_._ When corrected for the background signal (the average value between 127.5 and 126.5 ky BP), the δ^13^C values of single and pooled tests reveal a strong vertical depth gradient, with the bottom waters, intermediate depth waters, thermocline, and the mixed layer showing a Δδ^13^C value of −7.25‰, −4.66‰, −3.01‰, and −3.94‰, respectively (*SI Appendix*, Fig. S4).

We conducted δ^13^C and δ^18^O mass balance calculations, shell weight analyses of individual tests of *G. ruber* pink, and scanning electron microscope (SEM) image analysis of *G. ruber* pink and *U. peregrina* to test whether these anomalously low δ^13^C values resulted from diagenetic alteration of the primary foraminiferal calcite (*SI Appendix*, Figs. S5–S7). Our mass balance calculations (*SI Appendix*, Fig. S5) indicate that a postdepositional diagenetic calcite mass of 342 μg with δ^13^C and δ^18^O values of −6.99‰ and 0.18‰, respectively, would need to be added to a single *G. ruber* pink test (300 μm to 350 μm) with an average mass of 20 μg and δ^13^C and δ^18^O of 1.06 to 2.95‰ in order to explain the observed δ^13^C and δ^18^O values of −1.24‰ and −2.63‰, respectively. However, our weight measurements of individual tests of *G. ruber* pink (300 μm to 350 μm) show a normal distribution with a mean value of only 19.6 ± 4.5 μg (*n* = 42) (*SI Appendix*, Fig. S6), far less than the expected weight (342 μg) of a single test if affected by diagenesis. Consistent with results of shell weight measurements, high-resolution SEM images of *U. peregrina* and *G.* pink shells do not reveal any indication of postdepositional calcite precipitation (*SI Appendix*, Fig. S6). In distinct contrast to the δ^13^C data, the δ^18^O values of multispecies foraminifers fail to display any uniform trends, with some showing decreasing and others increasing δ^18^O values. A strong diagenetic imprint can be expected to affect both δ^13^C and δ^18^O trends. On the basis of the multiple- and single-test analyses (*SI Appendix*, Figs. S5–S7), postdepositional alterations can be ruled out as the cause for the anomalously low δ^13^C values.

Similarly, several observations are inconsistent with the possibility that the anomalously low δ^13^C values resulted from increased primary and export productivity and enhanced demineralization of organic materials. At a time of reduced riverine runoff (weakened West African monsoon), as indicated by increased δ^18^O in the mixed layer (Fig. 2*B*), a possible source of increased nutrients could possibly be from an increase in the upwelling of nutrient-rich deeper waters. However, such a process is not supported, through lack of attendant cooling in the mixed layer (Fig. 2*C*). Similarly, a lack of any distinct and large increase in benthic foraminiferal Ba/Ca and Cd/Ca, refractory nutrients, and elevated B/Ca is inconsistent with increased export productivity and enhanced degradation of organic matter as the source of the negative δ^13^C anomaly (Fig. 4).

The episode of anomalously low δ^13^C values occurred, within age model uncertainties, during an interval of a weakened AMOC (Figs. 3 and 4) (26, 28–30). Associated with this weakened ocean circulation, benthic foraminiferal δ^13^C decreased, on average, to −0.6‰ in North Atlantic Deep Water (30). Advection of South or North Atlantic deep waters with δ^13^C values of ∼−0.6‰ (30, 31) cannot explain the extreme δ^13^C values up to −3.8‰ and −8.67‰ in pooled and single foraminiferal analyses, respectively (*SI Appendix*, Fig. S8). Furthermore, an absence of substantially elevated benthic foraminiferal Ba/Ca and Cd/Ca is inconsistent with advection of refractory nutrient-rich waters to intermediate depths in the equatorial Atlantic (Fig. 4).

## Methane Hydrate Dissociation due to Strong Intermediate Water Warming

The most plausible cause of the anomalously low δ^13^C values was massive destabilization of methane hydrates in shallow subsurface marine sediments and ensuing methane flux and methane oxidation that affected the entire water column. Formed by upward migration and diffusion of methane via faults and other conduits, methane hydrate deposits are known to be widely distributed in shallow subsurface sediments of the continental margins of the Gulf of Guinea (32–35). An extensive sediment coring investigation on the Nigerian continental margin (within the Gulf of Guinea) demonstrated that about 2.5% of 826 sediment cores contained methane hydrates in the form of disseminated nodules within muddy sediments only 1.4 m to 3 m below the seafloor, or as massive methane hydrates 5 m to 6 m below the seafloor (33–35). Moreover, all sediment cores collected from water depths between 500 and 1,000 m contain massive gas hydrate deposits (33–35). The δ^13^C values of Gulf of Guinea methane hydrates range from −63 to −117‰, with an average value of −78.5‰, indicating a biogenic origin of the methane formed from organic matter decomposition under anoxic conditions (33). Methane hydrate destabilization occurs due to changes in gas saturation in the pore water, slope failures, pressure decrease due to sea level fall, and/or temperature increase (1, 2, 4, 5). With global sea level ∼3 m to 5 m higher than the present day (36, 37), a methane hydrate destabilizing role of sea level change can be ruled out during the Eemian.

**Fig. 5.**
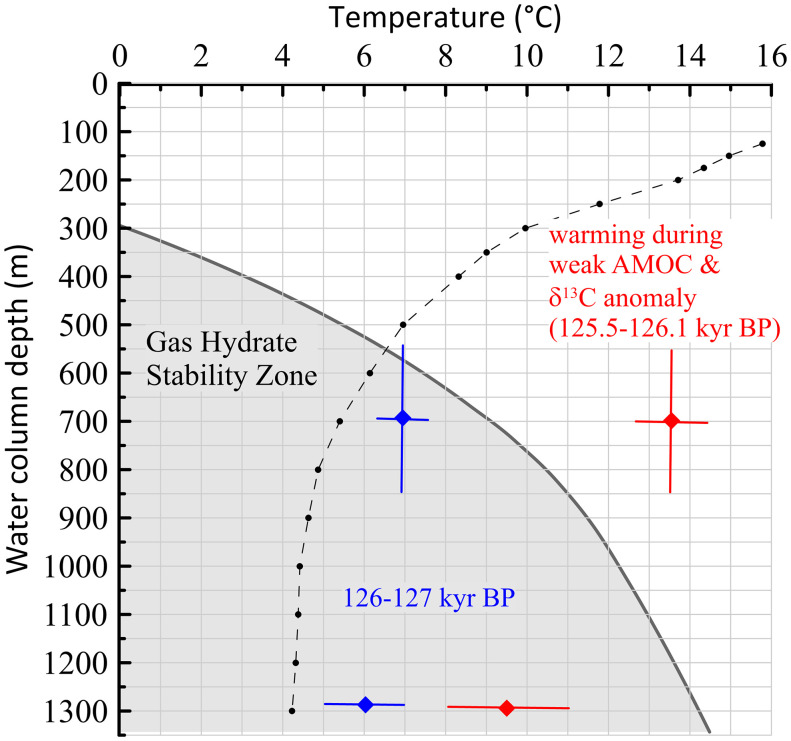
Warming of the intermediate water depth during meltwater-induced weakening of the AMOC and gas hydrate stability zone in the Gulf of Guinea is indicated by the gray shaded area (32, 33). Modern temperature profile of Golf of Guinea water column at 9.5°E/2.5°N is indicated by the black dash-dotted line (World Ocean Circulation Experiment (WOCE) gridded climatology data). Blue and red dots indicate temperature reconstruction of intermediate (700 ± 150 m) and bottom (1,295 m) water prior to and during the AMOC weakening and carbon isotope anomaly, respectively.

Strong evidence for the warming of intermediate waters that significantly exceeds the regional methane hydrate stability is provided by two independent proxy datasets (Figs. 4 and 5). Between the onset (126.5 ky BP) and peak (125.5 ky BP) of the δ^13^C anomaly, δ^18^O of *G. crassaformis* exhibits a remarkably large decrease of 1.5‰ (Fig. 4). In the absence of any large salinity decrease, this indicates an exceedingly large temperature rise of 6.8 °C of intermediate waters at ∼700-m water depth. In support of this, Mg/Ca-based calcification temperature reconstructions of *G. crassaformis* indicate that water temperature increased from 7.2 ± 0.8 °C at 126.9 ky BP to 126.3 ky BP to 13.6 ± 1.4 °C at 125.4 ky BP to 126.1 ky BP. Thus, the timing and magnitude of this intermediate water warming of 6.4 °C, as suggested by the Mg/Ca data, is consistent with the 6.8 °C rise inferred from the δ^18^O record. Such a high intermediate water temperature of 13.6 °C during the δ^13^C anomaly implies that, even if the average habitat depth of *G. crassaformis* had shallowed by 200 m or deepened by 500 m relative to its average habitat depth of 700 m (24), the magnitude of this warming would have significantly exceeded the threshold of the regional methane hydrate stability field for water depths between 500 and 1,200 m (Fig. 5) (32–35).

Although influenced by factors other than calcification temperatures (38, 39), our Mg/Ca analyses in tests of epibenthic foraminifers *C. wuellerstorfi* indicate that bottom water temperature (BWT) at 1,295 m increased from 4.8 °C at 126.7 ky BP to 11.8 °C at 125.2 ky BP, an increase of 7 °C concomitant with the timing of the δ^13^C anomaly (Fig. 4). Although the Mg/Ca-based estimated BWT of 11.8 °C at a seafloor depth of 1,295 m is actually within the methane hydrate stability zone (Fig. 5), the δ^13^C data of infaunal and epibenthic foraminiferal strongly indicate an isotopic signature of DIC most likely influenced by methane oxidation. Thus, it is possible that our BWT estimation at a seafloor depth of 1,295 m actually has significantly underestimated the magnitude of seafloor warming. Another possible, but related, process involving slope instability and methane hydrate deroofing is discussed below.

The major seafloor temperature increase, as shown by the Mg/Ca data, is also qualitatively corroborated by a decrease in δ^18^O of up to 0.9‰ in tests of *C. wuellerstorfi* (*SI Appendix*, Fig. S2). However, we note that this decrease in δ^18^O of *C. wuellerstorfi* within the δ^13^C anomaly likely resulted from a combination of both increased temperatures and δ^18^O changes in bottom waters, as discussed below. In conjunction with the BWT rise, the δ^18^O values of pooled tests (shells) of the infaunal benthic foraminifers *C. pachyderma* and *U. peregrina* exhibit an increase of 0.3 to 0.2‰ at the time when seafloor temperatures were actually increasing. This digression in values is most likely explained by the effects of interstitial waters associated with the dissociation of methane hydrates which are enriched in the heavier oxygen isotope (40) and in Ba (41). In contrast to the Cd/Ca, the Ba/Ca records exhibit a slight Ba increase relative to the intervals immediately before and after the δ^13^C anomaly. Similar to the δ^18^O trends exhibited by infaunal foraminifers, we suggest that this modest increase in Ba also likely resulted from release of gas hydrate waters in addition to sulfate reduction.

The ΔCO_3_^2-^ record, inferred from B/Ca analyzed in *C. wuellerstorfi*, did not change significantly during the carbon isotopic anomaly. Yet a decline in bottom water CO_3_^2−^ can be expected if only input of methane oxidation is considered. This observed lack of a CO_3_^2−^ decline may be explained by associated alkalinity change concomitant with CO_2_ inputs. Methane oxidation in the sediments would make porewater more acidic, thereby dissolving carbonates. This, in turn, would raise porewater alkalinity and bottom water alkalinity through diffusion and increase in CO_3_^2−^, counteracting any effect caused by the increase in CO_2_. Although future work is required to better explain reasons for a lack of CO_3_^2−^ changes, our documented role of methane oxidation is well supported by the δ^13^C record. Compared to CO_3_^2−^, δ^13^C changes may be more sensitive to methane releases, because methane has such negative δ^13^C values, compared with alkalinity which would have negligible effect on δ^13^C values. Thus, the observed δ^13^C decline throughout the water column is clearly consistent with massive methane release and oxidation.

Overall, our multispecies δ^18^O record representing from surface waters down to the seafloor (1,295 m) in combination with the temperature determinations based on Mg/Ca of *G. crassaformis* and *C. wuellerstorfi* indicates a pronounced water column warming between 400 m and the seafloor, with the greatest warming at ∼700 ± 150 m (Figs. 4 and 5 and *SI Appendix*, Fig. S2). Significantly, this closely matches to the center of the water depth range (500 m to 1,000 m) of shallow, subsurface methane hydrate deposits on the Nigerian continental margin (33). Our observations demonstrate the action of a strong positive climate warming feedback process. The global warming during the early part of the penultimate interglacial would have enhanced deglaciation of the Greenland ice sheet (36, 42, 43), with resulting increased meltwater input into critical areas of the North Atlantic, as documented by increased ice-rafted detritus (IRD) (26, 28). This fresh water input would, in turn, have weakened AMOC (26, 28–30). We suggest that the combined effect of a weakened AMOC and elevated sea surface temperature of the equatorial Atlantic (20) reduced the advection of cold water from high latitudes and enhanced downward heat diffusion, respectively, leading to significant warming of intermediate waters. This, in turn, potentially triggered further atmospheric warming by release of methane due to the major destabilization of shallow subsurface methane hydrate.

We suggest that two main processes were involved in this methane release. First, in the depth range where warming exceeded the regional temperature stability field of methane hydrates (Fig. 5), downward heat diffusion into the fairly shallow subsurface methane hydrates below loosely compacted sediments triggered methane hydrate dissociation. The lack of any substantial lag between the peak warming of the intermediate waters and the peak of the carbon isotope anomaly (Fig. 4 *A* and *B*) strongly indicates that the bulk thermal diffusivity of Gulf of Guinea sediments was sufficiently large and rapid and the enhanced downward heat diffusion was sustained for an extended period to cause methane hydrate dissociation, an endothermic reaction (44), without any significant delay, and which lasted for several hundred years. This indicates a direct and dynamic link between centennial- to millennial-scale Quaternary climate oscillations and dissociation of shallow subsurface methane hydrates. Our results provide a paleoclimate perspective that we believe will be helpful in setting constraints for the modeled hydrate dissociation timescale (5). Second, it is likely that the methane hydrate dissolution also caused slope instability, failures, and deroofing of sediment packages, directly exposing methane hydrates to conditions not conducive for methane hydrate stability and preservation, hence amplifying the process of methane hydrate dissociation (10, 45). This scenario helps reconcile the imprint of methane hydrate dissociation at a seafloor depth of 1,295 m with BWTs that fall within the temperature stability field of methane hydrates (Fig. 5). Alternatively, it is possible that our reconstruction of seafloor temperature at depths as much as 1,295 m significantly underestimates actual bottom water warming. These two interrelated processes for destabilization led to methane hydrate dissociation, methane release, and its oxidation throughout the entire water column. The oxidation of methane with its extremely low δ^13^C values (−63 to −117‰) (33) changed the carbon isotope signature of the DIC, as recorded in foraminiferal tests that calcified at different water column and seafloor depths (Figs. 1 and 3).

Our findings imply that the methane oxidation across the entire water column and potential methane release to the atmosphere must have been a significant and widespread process, as reflected by the anomalously low δ^13^C values inferred for the uppermost water column (Fig. 2). However, it has been argued that, because of low permeability of sediments, structural traps, and the anerobic oxidation of methane in sediment sequences, upward methane escape to the mixed layer and atmosphere is insignificant (2). These factors quite likely limit upward flux of methane stemming from dissociation of stratigraphic-type methane hydrates in the absence of significant bottom warming. However, the impact of these limiting factors on shallow subsurface methane hydrates imbedded in fairly loose sediments is likely insignificant. Moreover, sulfate reduction, which is considered to be coupled with anerobic oxidation of methane by a microbial consortium (46), is currently recorded in the Gulf of Guinea below a sediment depth of 6 m to 3 m (32, 47). This is below the depth of occurrence of most disseminated methane hydrate nodules (33–35). Results of a modeling investigation suggest that anerobic oxidation of methane in sediments would effectively prevent the upward escape of methane into the water column after ∼60 y, a modeled time the anerobic microbial consortium needs to fully develop, following inception of a massive methane hydrate destabilization (48). The duration of the δ^13^C anomaly in multitaxa foraminifers (Fig. 3), indicating methane oxidation throughout the water column, is several hundred years, which is inconsistent with this model (48). We suggest that our evidence in support of methane oxidation in the seafloor sediments and upward through the water column of 1,300 m strongly implies that methane hydrate dissociation and resultant methane release was massive and widespread, having overwhelmed any methane escape-limiting factors, including anerobic methane oxidation by a microbial consortium in the sediments.

We estimate the amount of oxidized methane (with an average δ^13^C value of −78.5‰) required to change the δ^13^C of DIC of the mixed layer from 1.06‰ (average value prior to the event) to −1.58‰, as inferred from δ^13^C of *G. ruber*, for a sea water volume of 1,500 km^3^ and DIC of 2,100 μmol/kg (for details, see *SI Appendix*, Fig. S8). While the choice of DIC concentration between 2,000 and 2,300 μmol/kg has only a small effect on the outcome, the estimate of water volume has a major effect (*SI Appendix*, Fig. S8). Though arbitrary, a volume of 1,500 km^3^ (34 km × 34 km × 1.298 km) is rather conservative given the strong and temporally persistent δ^13^C imprint in the DIC recorded throughout the 1,295-m water column and the large area bathed by these intermediate waters in the vicinity of the core site. We obtain an estimate of a cumulative oxidized methane amount of 7.5 * 10^12^ g, which is equivalent to ∼11 * 10^9^ m^3^. This estimate is based on the average δ^13^C value of the most negative values that make 10% (*n* = 43) of the single *G. ruber*’s test measurements (*n* = 431). While this approach captures the intensity of methane oxidation in the water column, single foraminiferal analysis does not reflect the entire episode of the anomalously low δ^13^C values. On the other hand, the δ^13^C time series of pooled shell analyses is well resolved and represents the entire anomalous episode. These data provide a cumulative methane oxidation of 5 * 10^12^ g (7.7 * 10^9^ m^3^), which is most likely an underestimation because each δ^13^C value represents a composite of the methane oxidation and background values. Recognizing these caveats, the minimum amount of oxidized methane released for an estimated water volume of 1,500 km^3^ ranges between 7.5 * 10^12^ and 5 * 10^12^ g. This calculation does not consider the methane that potentially escaped to the atmosphere. Given that intermediate waters in the Gulf of Guinea that dramatically warmed impinge on a large area with sediments containing shallow subsurface methane hydrates (33), the extent of methane hydrate dissociation, the release of the methane into water column, and its oxidation must have been substantial to have affected the entire water column. Furthermore, potential anerobic methane oxidation in the subsurface sediments remains unaccounted for. Taken together, it is likely that our calculation substantially underestimates the extent of methane hydrate dissociation and methane release into the water column. The challenge associated with such a potential scaling up of methane release would be best approached in the future through application of a comprehensive ocean carbon model.

While further studies are needed to determine the extent of methane hydrate destabilization during the weakened AMOC interval of the Eemian, the consequence of broad methane hydrate destabilization is increased atmospheric CH_4_ and CO_2_ concentrations. Taking age model uncertainties into consideration, during the peak in anomalously low carbon isotopes, the atmospheric CO_2_ and CH_4_ concentrations rose by 17 to 10 parts per million per volume and 20 parts per billion per volume, respectively (*SI Appendix*, Fig. S9) (49–51). Although the magnitude of this change varies between ice cores and analytical laboratories, the δ^13^C values of atmospheric CO_2_ declined by 0.3 to 0.4‰ coeval with the δ^13^C anomaly recorded in the Gulf of Guinea sediment sequence (*SI Appendix*, Fig. S9) (50, 52), indicating that a source with a significantly negative δ^13^C signature contributed to the increase of atmospheric CO_2_. Methane release and methane oxidation due to massive methane hydrate destabilization is the likely source. Quantifying the contribution of methane hydrate destabilization to this rise in atmospheric CO_2_ and CH_4_ concentration is, however, made more difficult because meltwater-induced AMOC weakening also would have led to increased atmospheric CO_2_ and CH_4_ concentrations due to enhanced ventilation of the Southern Ocean and strengthening of the Southern Hemisphere monsoon system, respectively (53–56). Furthermore, vertical gas diffusion in the Antarctic ice core record prior to compaction (57) most likely reduced the magnitude and blurred the temporal focus of a predicted atmospheric imprint due to methane hydrate destabilization.

## Strong Impact of Warm Mean Climate on Meltwater-Induced Intermediate Water Warming

Our study has revealed how the character of intermediate waters is so differently affected by meltwater-induced AMOC weakening at times of cold versus warm mean climatic states. The temperature of the tropical Atlantic intermediate waters increased by only 3 °C in response to strong and sustained meltwater-induced weakening of AMOC during the early phase of the last deglacial (19 ky BP to 14.6 ky BP), a time when tropical Atlantic sea surface temperatures were lower by 2 °C to 3 °C compared with the Early through Middle Holocene (20, 39). Similarly, the warming of the upper intermediate waters (∼500 m to 700 m) during the late phase of penultimate deglaciation appears relatively weaker and the SST slightly lower compared to the Eemian (Figs. 2 and 4), although the meltwater-induced AMOC weakening was strong, as judged from records of the IRD (Fig. 4), benthic foraminiferal δ^13^C, and planktonic foraminiferal δ^18^O in North Atlantic sediments (25, 26, 29). In contrast, intermediate water warming was much higher (6.4 °C to 6.8 °C) in response to a relatively brief and modest weakening of AMOC during the Eemian when tropical sea surface temperatures were 1.5 °C to 2 °C warmer than the early Holocene (20). Consistent with simulation results (6), we suggest that weakened advection of cold, high-latitude waters had the effect of strengthening the influence of downward diffusive heat from warm surface waters, leading to a large temperature increase in intermediate waters. This dramatic temperature rise, in turn, triggered massive methane hydrate destabilization. A major implication of this study is that, under warmer climatic conditions, the impact of AMOC weakening on the temperature of intermediate waters is exceptionally strong.

## Conclusions

We have investigated a sediment sequence from the eastern equatorial Atlantic using a wide range of environmental proxies at high chronological resolution. The records reveal an episode of major destabilization of methane hydrates during the early part of the penultimate warm period (126 ky BP to 125 ky BP). This episode of methane hydrate dissociation was caused by an extraordinarily large warming (6.8 °C) of intermediate waters causally linked to the meltwater-induced weakening of AMOC during ongoing climatic warming. The duration of this event and its magnitude indicate an extended and significant methane hydrate dissociation episode. With accelerated melting underway in Northern Hemisphere ice sheets and resultant meltwater input into the subpolar North Atlantic, this study demonstrates that processes at the onset of the penultimate warm episode can serve as an analog for how meltwater-induced AMOC weakening significantly amplifies the warming of intermediate waters and, in turn, destabilizes shallow subsurface methane hydrate deposits. Current studies that are investigating the stability of shallow subsurface methane hydrates assume warming of intermediate waters of only 1 °C to 3 °C, and, as a result, conclude a limited impact on their stability. Robust indications of a steadily weakening AMOC and the amplifying effect of weak AMOC on the warming of intermediate waters, as documented here, require simulation scenarios that consider much greater warming of intermediate waters.

## Materials and Methods

### Setting.

This study focuses on a sediment core(MD2707) recovered from the Gulf of Guinea, eastern equatorial Atlantic (2°30.11’N, 9°23.68’E, 1,295-m seafloor water depth) (Fig. 1). The surface water above the core site is strongly influenced by runoff of the Niger and Sanaga Rivers that drain a large part of the West African monsoon region (*SI Appendix*, Figs. S1).

### Age Model.

The age model of the target MD2707 sediment section was established by tuning the δ^18^O_G. ruber_ record to the δ^18^O_stalagmite_ from Sanboa Cave in China (58) (*SI Appendix*, Fig. S1). The rationale for this tuning approach is based on two key observations. Firstly, the δ^18^O of surface water over the MD2702 site is strongly influenced by the large amount of fresh water input from rivers that drain large part of West African monsoon areas (*SI Appendix*, Fig. S1). Therefore, the δ^18^O_surface water_ record of MD2707, which is calculated using the δ^18^O of *G. ruber pink*, serve as a proxy for changes in the West African monsoon (20, 59, 60). Secondly, the radiocarbon-dated sections of MD2707 (0.3 ky BP to 38 ky BP) show that changes in the West African monsoon (20, 61) synchronously covaried, within the dating uncertainties, with changes in the East Asian monsoon, as recorded in stalagmites from Chinese caves (62) (*SI Appendix*, Fig. S1). On the basis of the assumption that the millennial-scale covariation of West African and East Asian monsoons holds through Termination II and Last Interglacial, we argue that our tuning approach is the best solution to obtain a robust age model for the benthic foraminiferal record of the targeted MD2707 sediment section.

### Carbon and Oxygen Isotope Analysis.

We analyzed carbon (^13^C/^12^C) and oxygen isotope (^18^O/^16^O) ratios, expressed as δ^13^C (‰, Vienna Pee Dee Belemnite (VPDB) )= {[(^13^C/^12^C)_sample_/(^13^C/^12^C)_standard_] − 1} * 10^3^ and δ^18^O (‰, VPDB) = {[(^18^O/^16^O)_sample_/(^18^O/^16^O)_standard_] − 1} * 10^3^, in pooled and single tests of *G. ruber* (pink) *sensu stricto* (shell size: 250 μm to 300 μm), *N. dutertrei* (300 μm to 400 μm), *G. truncatulinoides* (300 μm to 400 μm), *G. crassaformis* (300 μm to 400 μm), *C. wuellerstorfi* (>300 μm), *C. pachyderma* (>300 μm), and *U. peregrina* (>300 μm). For core-top samples, we analyzed *Cibicides* spp. (Fig. 1). The pooled samples consisted of 25 to 30 individuals of *G. ruber sensu stricto* (pink) and *N. dutertrei* and 13 to 16 individuals of all the other species were used for each analysis. The pooled foraminiferal tests were gently crushed between glass plates and checked for infills. The δ^18^O and δ^13^C analyses were carried out using a stable isotope ratio mass spectrometer (Thermo Scientific MAT253) online coupled to a sample preparation devise (Kiel IV) at the Department of Earth Science, University of California, Santa Barbara (UCSB). The external analytical uncertainty of δ^18^O and δ^13^C analysis in single and pooled foraminiferal shells is 0.07‰ and 0.03‰, respectively, and was assessed by repeated measurements of the carbonate standard NBS 19.

### Trace Element Analyses.

The cleaning protocol of gently crushed tests of *C. wuellerstorfi* (>300 μm) for the trace element analyses includes NaOH-buffered hydrogen peroxide and anhydrous hydrazine/ammonium citrate/ammonium hydroxide treatments as described in Martin and Lea (63). Trace elements were analyzed using a quadrupole inductively coupled mass spectrometer (ICP-MS) and analytical methods as described in Yu et al. (64). Analytical reproducibility, assessed by analyzing consistency standards matched in Mg/Ca and B/Ca ratios to dissolved foraminiferal solutions, is 0.39% (±0.008 mmol/mol) and 0.62% (±0.94 mmol/mol), respectively. The accuracy is 1.46% and 0.17% for Mg/Ca and B/Ca, respectively. Mg/Ca analyses in single tests of *G. crassaformis* (300 μm to 400 μm) were carried out using Laser Ablation ICP-MS at UCSB, using a Photon Machines Excite 193-nm laser coupled to an Agilent 7700x quadrupole ICP-MS using similar instrument conditions to those described in Kylander-Clark (65). Five to 10 tests of *G. crassaformis* per sample were selected, and three analyses per test were used to measure Mg, Fe, Mn, and Al, using a 50-µm spot at 10 Hz for 60 s. Data were processed with Iolite v3 (66) using NIST610 as the primary reference material and ^44^Ca as the internal standard, assuming 40 wt% Ca. Within each sample, all analyses with a Mn/Ca value less than 0.35 mmol/mol were pooled to obtain a mean value. The reproducibility of the measurements, as assessed using a nanoparticulate pressed calcite powder tablet, is 4%, which is smaller than the SD of the pooled values.

### Conversion of Trace Elements into Temperature Estimates.

For the conversion of Mg/Ca and B/Ca to estimates of temperature and ΔCO^2−^_3_, we used the following equations: ΔCO^2-^_3_ (μmol/kg) = (1.14 ± 0.048) * B/Ca + 177.1 (67), T(°C) = [Mg/Ca − (0.0087 ± 0.0007) * ΔCO^2−^_3_ − (0.82 ± 0.03)]/(0.056 ± 0.011) for *C. wuellerstorfi* (38), T(°C) = [LN(Mg/Ca) + 0.036 * (S-35) − 0.73 × (pH-8)]/0.061 (68) S = 34.8, pH = 7.95 for *G. crassaformis*.

### Mass Balance Calculation.

To assess a potential diagenetic effect, we used the following equations and assumptions:

$$\delta^{13}\text{C}=\delta^{13}{\text{C}}_{\text{background}}*{\text{Mass}}_{\text{background}}+\delta^{13}{\text{C}}_{\text{diagenetic}}*{\text{Mass}}_{\text{diagentic}}$$

$$\delta^{18}\text{O}=\delta^{18}{\text{O}}_{\text{background}}*{\text{Mass}}_{\text{background}}+\delta^{18}{\text{O}}_{\text{diagenetic}}*{\text{Mass}}_{\text{diagentic}}.$$

The δ^13^C_background_, δ^18^O_background_ and Mass_background_ values are estimated using average values of the time interval (126 ky BP to 128 ky BP) prior to the anomalously low δ^13^C event. The δ^13^C_diagenetic_ and δ^18^O_diagenetic_ values are approximated by the difference between δ^13^C and δ^18^O of *U. peregrina* during the anomalously low δ^13^C event and an average value of the time interval (126 ky BP to 128 ky BP) prior to the anomalously low δ^13^C event: δ^13^C_diagenetic_ = δ^13^C_U. peregrina_ (event) − δ^13^C_U.peregrina_ (background); δ^18^O_diagenetic_ = δ^18^O_U.peregrina_ (event) − δ^18^O_U. peregrina_ (background). The δ^13^C_diagenetic_ and δ^18^O_diagenetic_ are −6.99‰ and 0.18‰, respectively.

For *G. ruber* pink, the δ^13^C_background_, δ^18^O_background_, and Mass_background_ are 1.06‰, −2.95‰, and 20.5 μg, respectively. Mass (μg) = Mass_background_ + Mass_diagenetic._ The data depicted in *SI Appendix*, Fig. S3 are obtained by calculating the amount of the diagenetic material that is needed to explain a range of δ^13^C and δ^18^O values. *SI Appendix*, Fig. S3 demonstrates that a diagenetic material of 342 μg in a single test of *G. ruber* is required to explain both the observed δ^13^C and δ^18^O of −1.24‰ and −2.63‰, respectively.

## Supplementary Material

Supplementary File

## Data Availability

All data presented in this study are available in the *SI Appendix* (Excel file), or on the website of the corresponding author (https://weldeab.geol.ucsb.edu/publications) (70).
