# Supplementary material for: Evidence for massive methane hydrate destabilization during the penultimate interglacial warming
Source: Proc Natl Acad Sci U S A. 2022 Aug 22;119(35):e2201871119. doi: 10.1073/pnas.2201871119 (PMC9436375; doi:10.1073/pnas.2201871119)
Supplement: Supplementary File [file pnas.2201871119.sapp.pdf]

Supplemental information

to

Evidence for massive methane hydrate destabilization during the penultimate interglacial warming

Syee Weldeab[1], Ralph Schneider[2], Jimin Yu[3,4], Andrew Kylander-Clark[1]

[1]Department of Earth Science, University of California, Santa Barbara, California 93106, USA

[2]Institute of Geosciences, Kiel University, 24118 Kiel, Germany

[3]Pilot National Laboratory for Marine Science and Technology (Qingdao), Qingdao, 266237, China

[4]Research School of Earth Sciences, The Australian National University, Canberra, Australia

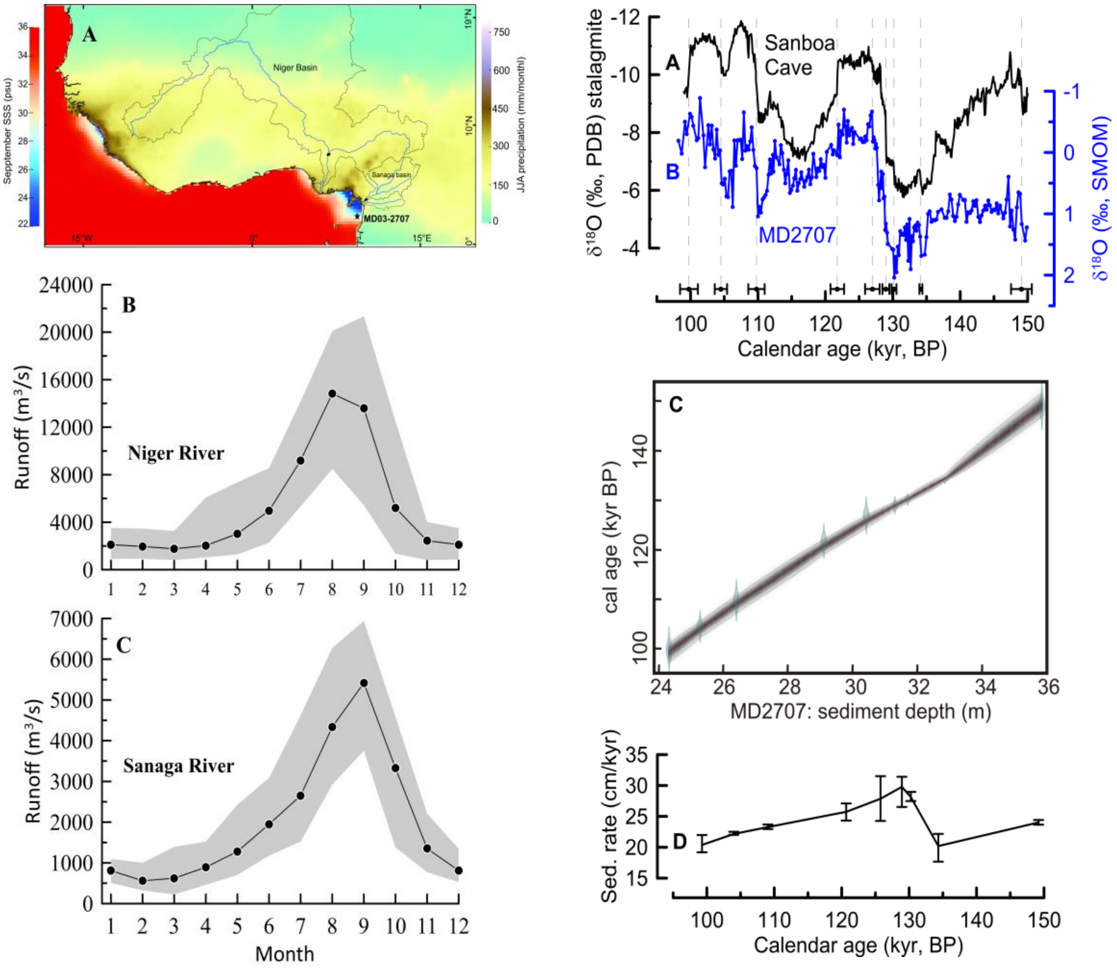

Figure S1: **Left and column**: West African Monsoon precipitation and it effect on the sea surface salinity of the Gulf of Guinea. A) monthly average of precipitation during the monsoon season (June-to-August). A 40-year average and upper and lower values of Niger River (B) and Sanaga River (B) runoff measured at Lokoja and Eda (black dot in the Figure S1A), respectively (The Global Runoff Data Centre, 56068 Koblenz, Germany, https://www.bafg.de/GRDC). **Right hand column**: Age model for MD03-2707 based on tuning the $\delta^{18}O$ record of *G. ruber* (MD03-2707) (A) to the $\delta^{18}O$ record Sanboa Cave stalagmite (B) (Cheng et al., 2009; Wang et al., 2008b). Vertical dotted lines indicate tuning points. The tie points of the age model are focused at the center of abrupt transitions in the stalagmite $\delta^{18}O$ record. C) Depth-age plot used to establish the age model for MD03-2707.

The uncertainty of the tie points is assessed as a propagative error resulting from the uncertainties (0.1–1.5 kyr) in the U-Th dating of the stalagmite and the range (0.1–0.5 kyr) with which a tie point can be shifted within the steep $\delta^{18}O$ changes (Figure 4). The age of the tie points and corresponding sediment depths are linearly correlated with an r^2 of 0.996. Shaded envelop indicates 1sigma and 2sigma uncertainties of the age model based on Bacon age model softtware (67, 68). The age model confidence interval (95%) for very sampling sediment depth is assessed using the Bayesian statistic software “Bacon” (67, 68) and varies between ±0.7 and ±1.6 kyr (Figure 4C). Sediment accumulation rate varies between 20 and 30 cm/kyr (Figure 4D). The elevated sediment accumulation rate (30 cm/kyr) during the penultimate deglacial is a persistent deglaciation feature that is also evident in the radiometrically dated last deglacial MD03=2707 record.

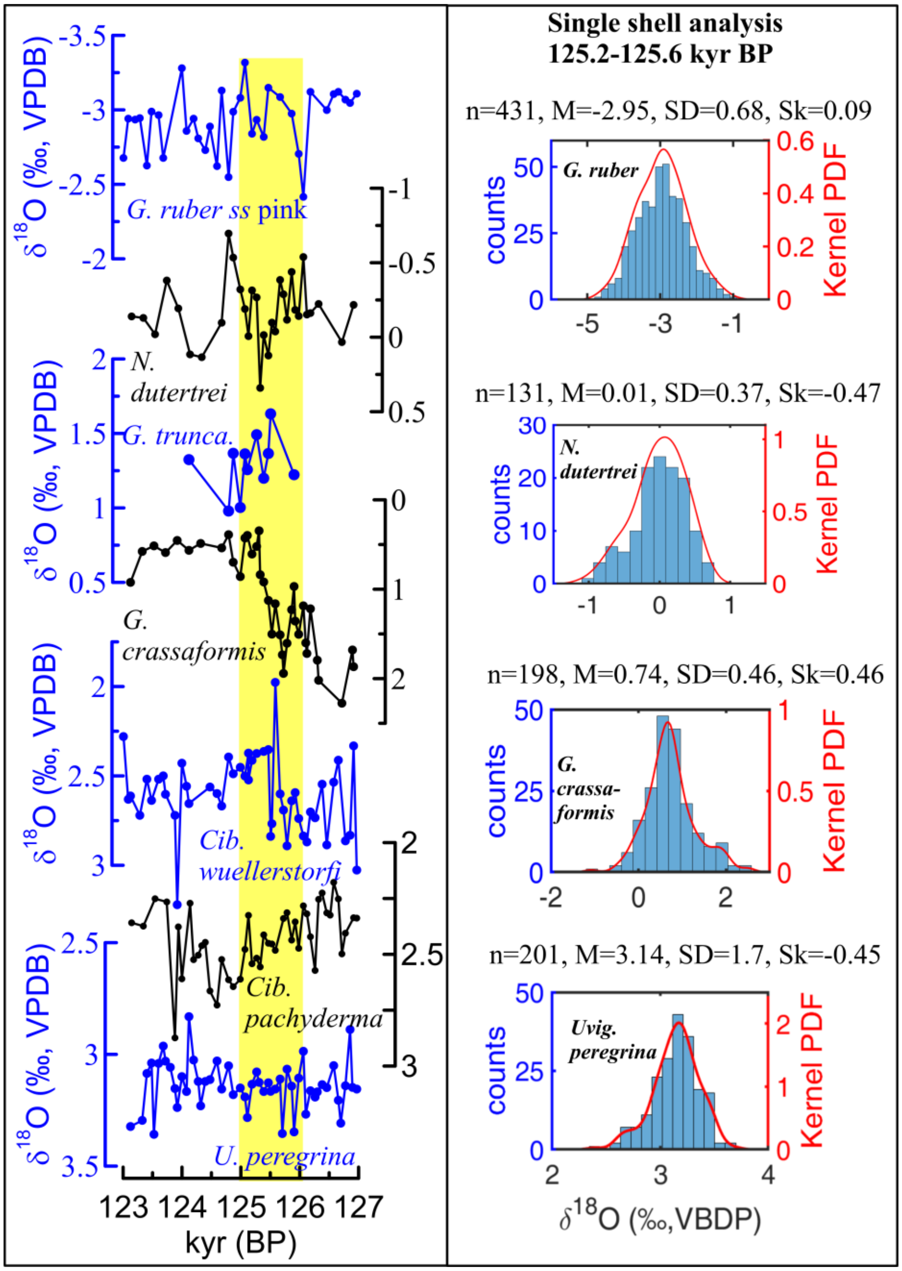

Figure S2: Left hand column shows $\delta^{18}O$ analyzed in pooled tests of multi-species benthic and planktonic foraminifers identified in the plot. Right hand column shows $\delta^{18}O$ analyzed in single tests of multi-species benthic and planktonic foraminifers identified in the plot.

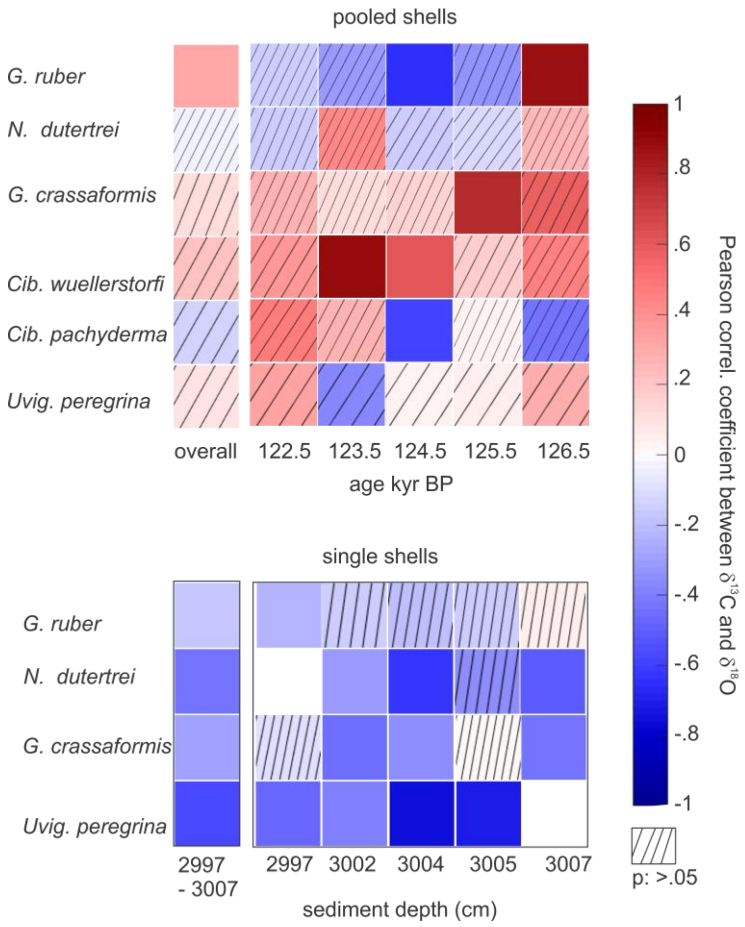

Figure S3: (Upper panel) Pearson correlation coefficient between δ^{18}O and δ^{13}C analyzed in pooled tests of benthic and planktonic foraminifers for the time-series shown in Figure 4 (δ^{13}C) and Figure S2 (δ^{18}O). The left column shows correlation efficient for the time window from 123 to 127 kyr BP. The rest columns show correlation coefficients for an average time of 1000 years centered at the show time point. Correlations with a p-value > 0.05 is indicated diagonal lines. Note that within the δ^{13}C anomaly (125.5 kyr BP) only the δ^{18}O and δ^{13}C of *G crassaformis* are significantly correlated. (Lower panel) Pearson correlation coefficient (R) between δ^{18}O and δ^{13}C analyzed in single tests of benthic and planktonic foraminifers from sampling depth within the δ^{13}C anomaly (125-to-126 kyr BP).

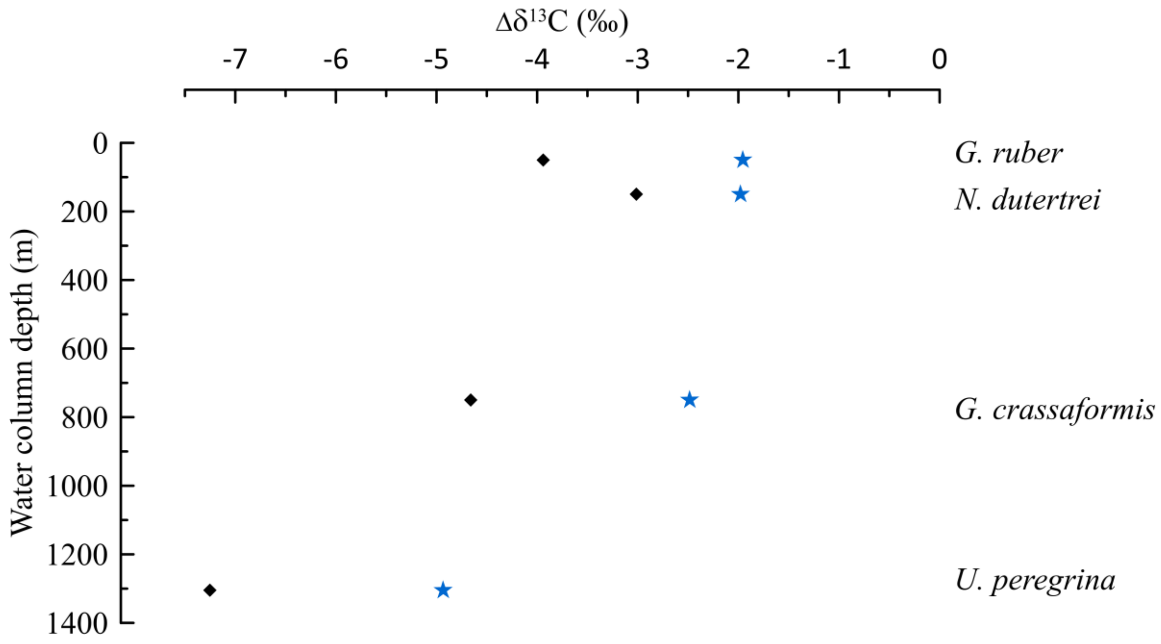

Figure S4: Difference between $\delta^{13}C$ of the anomalous interval and the background values of $\delta^{13}C$ (average value of pooled shells between 126.5 and 127 kyr BP). Black diamonds: difference between the lowest $\delta^{13}C$ of single test analysis and the average background value. Blue stars: average $\delta^{13}C$ values of 20% of the single test analysis and the average background value.

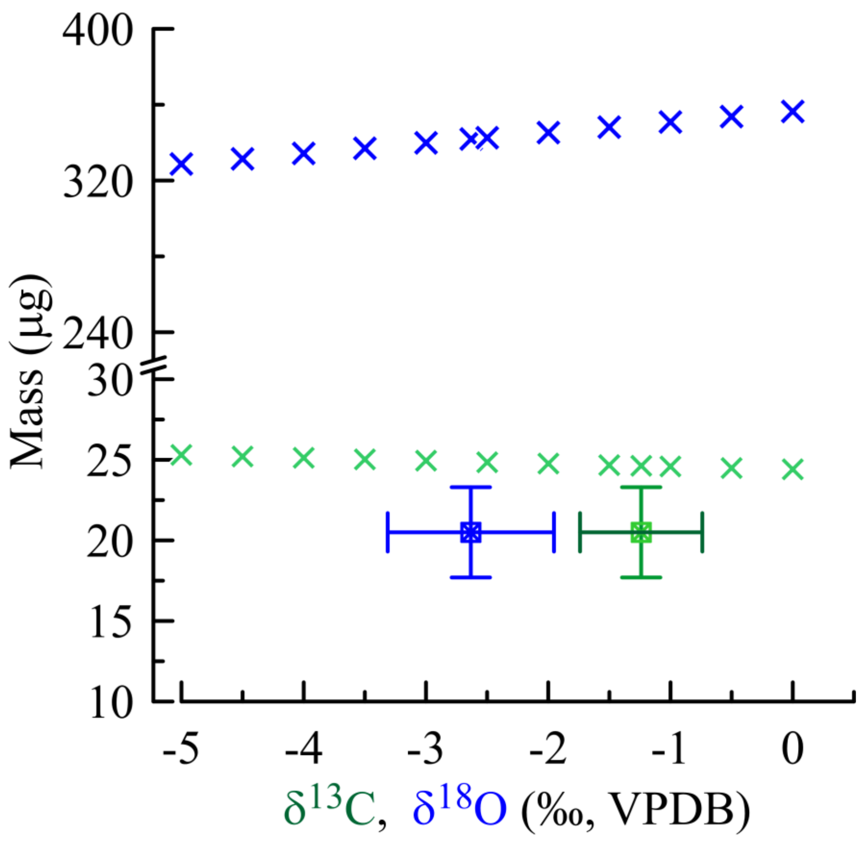

Figure S5: Amount of diagenetic material (Mass (μg)= $Mass_{background=20\ \mu g}$ + $Mass_{diagentic}$) required to obtain a range of $\delta^{13}C$ and $\delta^{18}O$ values in a single test of G. ruber. Modeled $\delta^{13}C$ and $\delta^{18}O$ data (symbols X and X) are obtained when average $\delta^{13}C$ and $\delta^{18}O$ of the 10% (n=21) of the single test analysis of *U. peregrina* with the most negative $\delta^{13}C$ values and the corresponding $\delta^{18}O$ values are used to calculate $\delta^{13}C_{diagenetic}$ and $\delta^{18}O_{diagenetic.}$ The rationale of this approach is to obtain a representative isotope signature of the event. Shown are also the average of $\delta^{13}C$ and $\delta^{18}O$ value of the 10% (n=43) of the single test analysis of *G. ruber* pink with the most negative $\delta^{13}C$ values and the corresponding $\delta^{18}O$ values. Horizontal and vertical bars indicate the standard deviation from the mean.

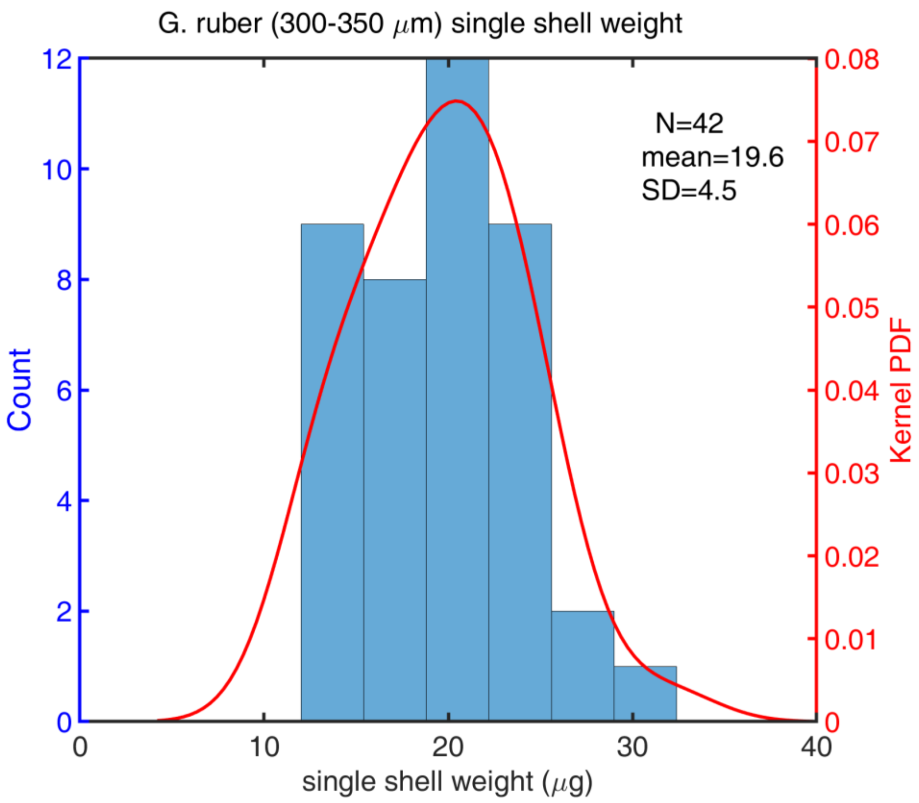

Figure S6: Histogram and Kernel probability density function (PDF) showing the weight distribution of single tests (n=42, mean value: 19.6 and standard deviation from the mean:4.5 μg) of *G. ruber* pink (300-350 μm) selected from the sampling depth 3007 cm within the $\delta^{13}C$ anomaly.

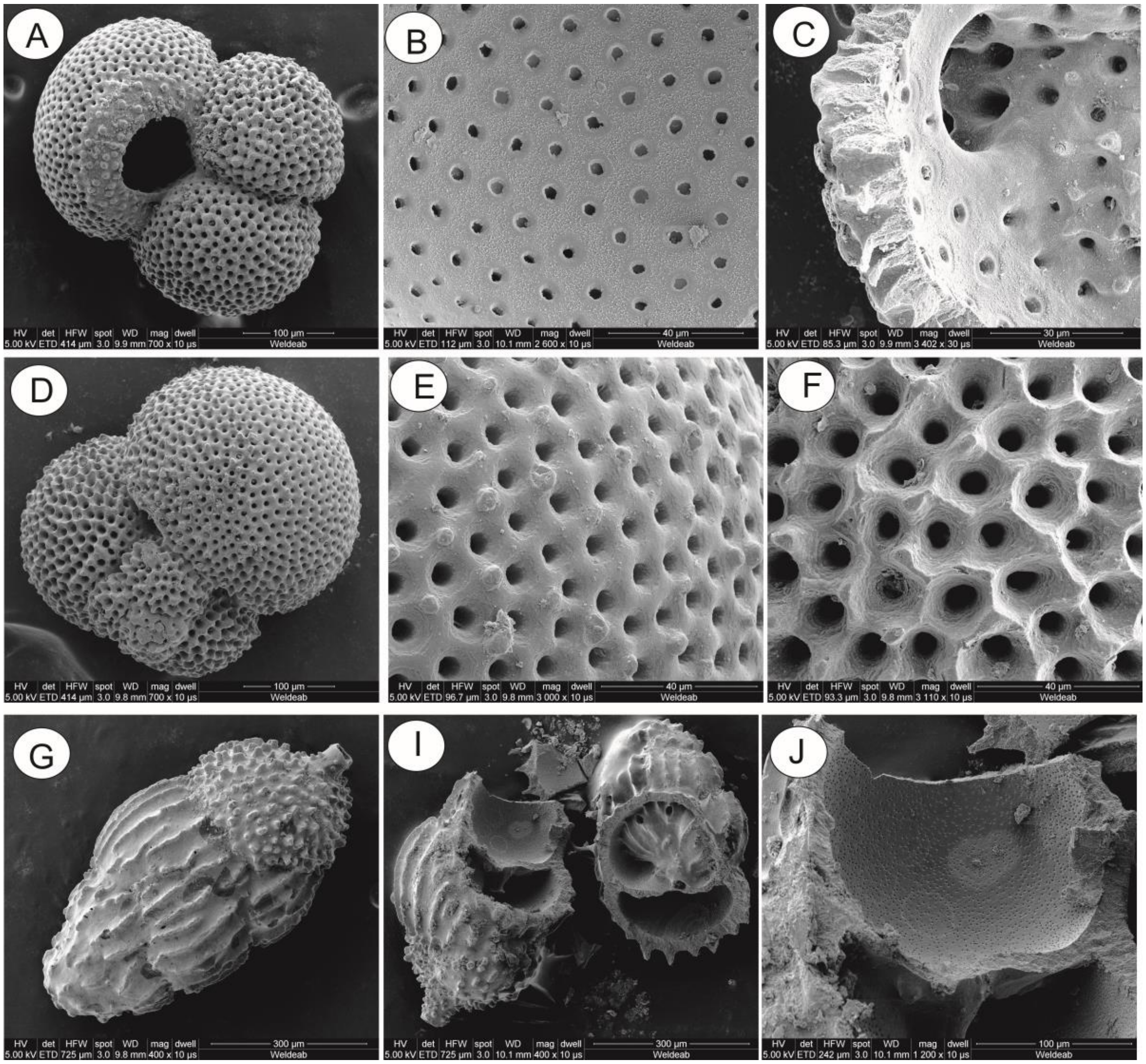

Figure S7: Scanning electron microscope images of tests and test fragments of *G. ruber* (A-F) and *Uvigerina peregrine* (G-J) from a sample within the interval of the $\delta^{13}C$ anomaly.

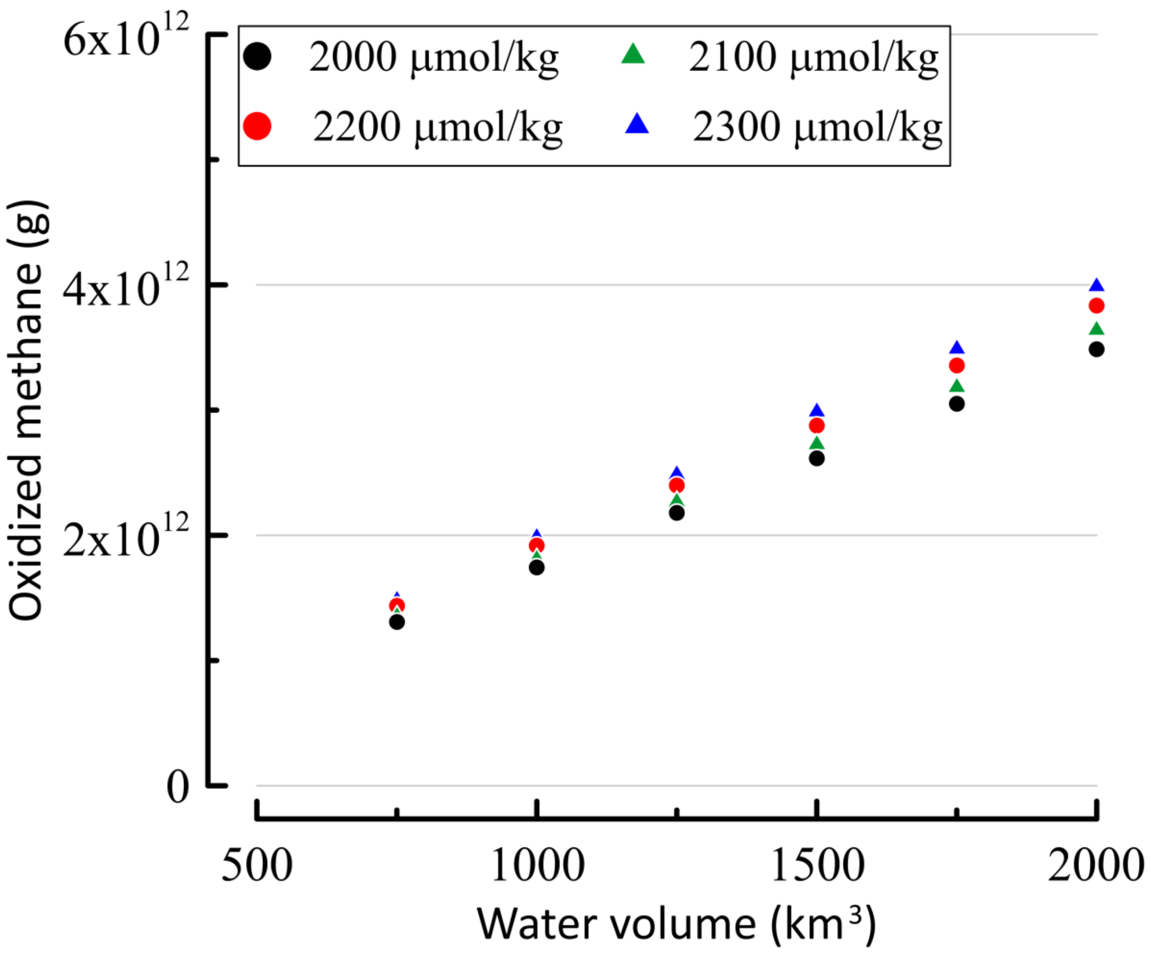

Figure S8: Modelled estimate of oxidized methane for a variable dissolved inorganic carbon and water volume that is affected by the methane flux and methane oxidation.

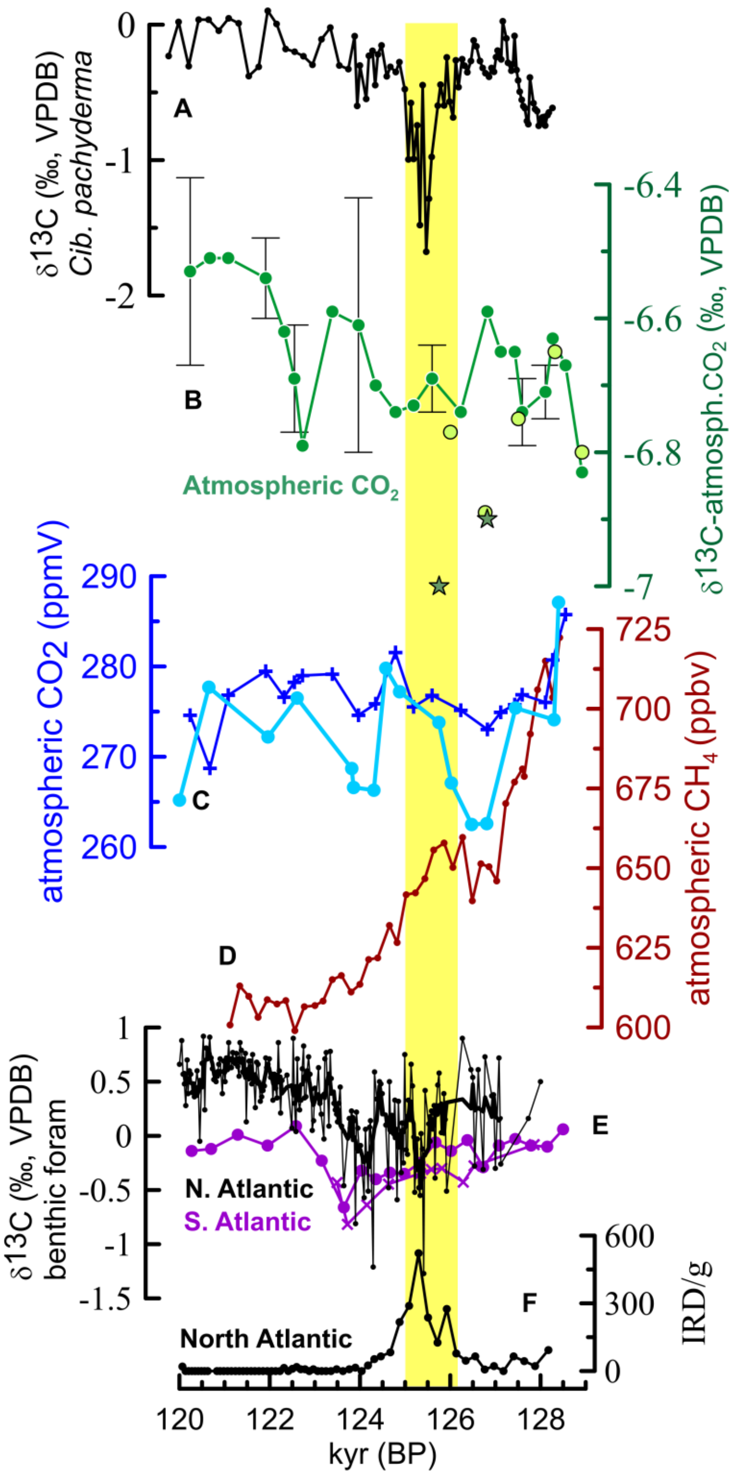

Figure S9: Changes in greenhouse gases and ocean circulation during the $\delta^{13}C$ anomaly. A) $\delta^{13}C$ time-series analyzed in pooled tests of *Cibicdes pachyderma (*this study). B) $\delta^{13}C$ of atmospheric CO2 (dark green circle: Dome C (47), light green circle: Talos Dome (47),

light green star: **Talos Dome** (51)). D) atmospheric CO_2 concentration (light blue: (46) and dark blue: (47). D) atmospheric methane concentration (48). E) Benthic foraminiferal $\delta^{13}C$ analyzed in a North Atlantic (black line) and South Atlantic (purple) core sediment (29, 30). F) Record of ice rafted detritus (IRD) in North Atlantic sediments (28) .

Sample	Latitude	Longitude	Sea floor depth (m)	sampling depth (cm)	Species	$\delta^{13}C$ (‰, VPDB)	$\delta^{18}O$ (‰, VPDB)	**depth binned $\delta^{13}C$ (‰, VPDB)**	**Stdv from the mean**
16876-1A	-0.33	9.02	27.00	0-1	Cib lobatulus	1.31	-0.51	**0.99**	**0.17**
16824-5A	4.77	-2.20	29.00	0-1	Cib mollis	1.16	-0.71		
16826-4A	4.95	-1.14	32.00	0-1	Cib lobatulus	1.03	-0.79		
16759-1A	8.65	-14.08	35.00	0-1	Cib lobatulus	0.93	-0.67		
16751-1A	10.08	-16.10	37.00	0-1	Cib lobatulus	0.88	-0.47		
16839-1A	5.95	1.14	39.00	0-1	Cib lobatulus	0.83	-0.64		
16847-1A	6.33	3.75	40.00	0-1	Cib lobatulus	1.33	-0.97		
16827-2A	4.65	-1.13	48.00	0-1	Cib mollis	1.21	-0.57		
16823-2A	4.67	-2.24	62.00	0-1	Cib lobatulus	1.08	-0.12		
16760-1A	8.48	-14.30	66.00	0-1	Cib lobatulus	0.76	-0.14		
16828-1A	4.42	-1.14	70.00	0-1	Cib lobatulus	0.99	0.11		
16877-1A	-0.34	8.94	72.00	0-1	Cib lobatulus	1.03	-0.19		
16783-1A	4.67	-8.90	75.00	0-1	Cib pseudoungerianus	0.95	0.05		
16878-2A	-0.34	8.90	96.00	0-1	Cib lobatulus	0.84	0.13		
16849-1A	6.17	3.70	99.00	0-1	Cib lobatulus	0.86	0.13		
16784-2A	4.58	-9.03	99.00	0-1	Cib lobatulus	0.86	0.31		
16829-2A	4.39	-1.15	100.00	0-1	Cib lobatulus	0.82	0.35		
16761-1A	8.37	-14.37	131.00	0-1	Cib mollis	1.28	0.40	**1.28**	
16830-1A	4.35	-1.15	201.00	0-1	Cib lobatulus	0.82	0.61	**0.65**	**0.17**
16813-?	5.00	-4.54	297.00	0-1	Cib pachyderma	0.48	1.51		
16762-2A	8.39	-14.40	302.00	0-1	Cib pachyderma	1.21	0.75		
16801-1A	4.52	-6.47	310.00	0-1	Cib mollis	0.38	1.39	**0.38**	
16820-1A	4.50	-2.30	445.00	0-1	Cib pachyderma	0.32	1.75	**0.35**	**0.11**
16816-1A	5.00	-4.52	475.00	0-1	Cib pachyderma	0.25	1.73		
16851-1A	6.07	3.65	495.00	0-1	Cib pachyderma	0.50	1.90		
16880-1A	-0.33	8.65	560.00	0-1	Cib pachyderma	0.27	2.06	**0.27**	
16819-2A	4.45	-2.33	631.00	0-1	Cib pachyderma	0.41	2.26	**0.40**	**0.08**
16754-1A	9.50	-16.62	646.00	0-1	Cib pachyderma	0.54	2.07		
16787-1A	4.47	-9.19	674.00	0-1	Cib pachyderma	0.48	2.24		
16802-1A	4.50	-6.47	691.00	0-1	Cib pachyderma	0.37	2.23		
16862-1A	3.54	6.48	698.00	0-1	Cib pachyderma	0.32	2.19		
16814-1A	4.98	-4.52	698.00	0-1	Cib pachyderma	0.30	2.02		
16763-1A	8.37	-14.43	701.00	0-1	Cib pachyderma	0.46	2.12	**0.51**	**0.12**
16843-1A	5.77	1.15	740.00	0-1	Cib pachyderma	0.34	2.20		
16818-1A	4.38	-2.37	749.00	0-1	Cib pachyderma	0.65	2.26		
16852-1A	6.07	3.64	770.00	0-1	Cib pachyderma	0.61	2.19		
16875-1A	-0.33	8.55	803.00	0-1	Cib pachyderma	0.41	2.31	**0.58**	**0.17**
16788-1A	4.43	-9.27	831.00	0-1	Cib pachyderma	0.75	2.45		
16815-2A	4.97	-4.52	913.00	0-1	Cib pachyderma	0.53	2.25	**0.63**	**0.08**
16832-1A	4.33	-1.14	920.00	0-1	Cib pachyderma	0.55	2.44		
16800-1A	4.47	-6.44	982.00	0-1	Cib pachyderma	0.68	2.45		
16863-2A	3.39	6.40	993.00	0-1	Cib pachyderma	0.73	2.32		
16874-1A	-0.34	8.47	997.00	0-1	Cib pachyderma	0.65	2.41		
16817-18	4.32	-2.38	1001.00	0-1	Cib pachyderma	0.63	2.57	**0.63**	**0.01**
16853-1A	6.05	3.63	1018.00	0-1	Cib pachyderma	0.62	2.37		
16764-1A	8.37	-14.43	1025.00	0-1	Cib pachyderma	0.64	2.82		
16806-1A	4.95	-4.55	1204.00	0-1	Cib pachyderma	0.57	2.53	**0.60**	**0.03**
16844-1A	5.72	1.15	1230.00	0-1	Cib pachyderma	0.64	2.47		
16833-1A	4.30	-1.15	1466.00	0-1	Cib pachyderma	0.86	2.70	**0.78**	**0.11**
16864-1A	3.15	6.28	1495.00	0-1	Cib pachyderma	0.86	2.57		
16756-1A	9.07	-17.00	1495.00	0-1	Cib pachyderma	0.62	2.94		
16809-2A	4.15	-2.47	1500.00	0-1	Cib pachyderma	0.77	2.64	**0.68**	**0.07**
16765-1A	8.32	-14.48	1500.00	0-1	Cib pachyderma	0.66	2.55		
16799-1A	4.44	-6.43	1505.00	0-1	Cib pachyderma	0.59	2.59		
16854-1A	6.02	3.60	1583.00	0-1	Cib pachyderma	0.71	2.64		
16873-1A	-0.33	8.30	1619.00	0-1	Cib pachyderma	0.73	2.64	**0.73**	
16869-1A	-0.20	6.00	1837.00	0-1	Cib robustus	0.63	2.62	**0.63**	
16845-1A	5.55	1.15	2007.00	0-1	Cib pachyderma	0.83	2.66	**0.82**	**0.01**
16845-1A	5.55	1.15	2007.00	0-1	Cib wuellerstorfi	0.81	2.54		
16845-1A	5.55	1.15	2007.00	0-1	Cib wuellerstorfi	0.83	2.61		

Table S1: $\delta^{13}C$ and $\delta^{18}O$ analyzed in *Cibicides spp* from Gulf of Guinea core top samples. Bold highlighted columns show the depth-binned mean $\delta^{13}C$ value and its standard deviation from the mean as shown in Figure 1. The binning interval is 100 m. Sea also SI Appendix (excel file).
